# MD2 contributes to the pathogenesis of perioperative neurocognitive disorder via the regulation of α5GABA_A_ receptors in aged mice

**DOI:** 10.1186/s12974-021-02246-4

**Published:** 2021-09-16

**Authors:** Wenqiang Zuo, Jianshuai Zhao, Jinming Zhang, Zongping Fang, Jiao Deng, Ze Fan, Yaru Guo, Jing Han, Wugang Hou, Hailong Dong, Feifei Xu, Lize Xiong

**Affiliations:** 1 Department of Anesthesiology and Perioperative Medicine, Xijing Hospital, Air Force Medical University, Xi’an, 710032 China; 2 Key Laboratory of Modern Teaching Technology, Ministry of Education, Shaanxi Normal University, Xi’an, 710062 China; 3 Department of Anesthesiology and Perioperative Medicine, Shanghai Fourth People’s Hospital Affiliated to Tongji University School of Medicine, Translational Research Institute of Brain and Brain-Like Intelligence Affiliated to Tongji University School of Medicine, Shanghai, 200434 China

**Keywords:** Perioperative neurocognitive disorder, Aged mice, MD2, α5GABA_A_R

## Abstract

**Background:**

Perioperative neurocognitive disorder (PND) is a long-term postoperative complication in elderly surgical patients. The underlying mechanism of PND is unclear, and no effective therapies are currently available. It is believed that neuroinflammation plays an important role in triggering PND. The secreted glycoprotein myeloid differentiation factor 2 (MD2) functions as an activator of the Toll-like receptor 4 (TLR4) inflammatory pathway, and α5GABA_A_ receptors (α5GABA_A_Rs) are known to play a key role in regulating inflammation-induced cognitive deficits. Thus, in this study, we aimed to investigate the role of MD2 in PND and determine whether α5GABA_A_Rs are involved in the function of MD2.

**Methods:**

Eighteen-month-old C57BL/6J mice were subjected to laparotomy under isoflurane anesthesia to induce PND. The Barnes maze was used to assess spatial reference learning and memory, and the expression of hippocampal MD2 was assayed by western blotting. MD2 expression was downregulated by bilateral injection of AAV-shMD2 into the hippocampus or tail vein injection of the synthetic MD2 degrading peptide Tat-CIRP-CMA (TCM) to evaluate the effect of MD2. Primary cultured neurons from brain tissue block containing cortices and hippocampus were treated with Tat-CIRP-CMA to investigate whether downregulating MD2 expression affected the expression of α5GABA_A_Rs. Electrophysiology was employed to measure tonic currents. For α5GABA_A_Rs intervention experiments, L-655,708 and L-838,417 were used to inhibit or activate α5GABA_A_Rs, respectively.

**Results:**

Surgery under inhaled isoflurane anesthesia induced cognitive impairments and elevated the expression of MD2 in the hippocampus. Downregulation of MD2 expression by AAV-shMD2 or Tat-CIRP-CMA improved the spatial reference learning and memory in animals subjected to anesthesia and surgery. Furthermore, Tat-CIRP-CMA treatment decreased the expression of membrane α5GABA_A_Rs and tonic currents in CA1 pyramidal neurons in the hippocampus. Inhibition of α5GABA_A_Rs by L-655,708 alleviated cognitive impairments after anesthesia and surgery. More importantly, activation of α5GABA_A_Rs by L-838,417 abrogated the protective effects of Tat-CIRP-CMA against anesthesia and surgery-induced spatial reference learning and memory deficits.

**Conclusions:**

MD2 contributes to the occurrence of PND by regulating α5GABA_A_Rs in aged mice, and Tat-CIRP-CMA is a promising neuroprotectant against PND.

**Supplementary Information:**

The online version contains supplementary material available at 10.1186/s12974-021-02246-4.

## Introduction

Perioperative neurocognitive disorder (PND), defined as cognitive impairment during the perioperative period, has been widely reported in clinical and animal studies in the past few decades. Patients suffering from PND require longer hospitalization, and some of them even fail to return to their domestic environment. Thus, this condition imposes a significant social and economic burden [1]. The number of patients suffering from PND is likely to increase gradually owing to the prolongation of the average life span of humans. Thus, the development of novel treatments for PND is urgently needed, as effective clinical therapies are lacking and the mechanisms underlying the pathogenesis of PND are still unknown.

Extensive studies have revealed that peripheral systemic inflammation resulting from operative trauma disrupts the integrity of the blood–brain barrier (BBB) and triggers an inflammatory response in the brain, which is widely recognized as a main contributor to the occurrence of PND [2]. Myeloid differentiation factor 2 (MD2), a protein that functions as a coreceptor of Toll-like receptor 4 (TLR4), is critical for the activation of the immune system and the release of inflammatory cytokines [3, 4]. Administration of lipopolysaccharide (LPS), a well-known activator of the MD2/TLR4 signaling pathway resulted in neuroinflammation and memory impairment in mice [5, 6]. In addition, a previous study showed that mice deficient in myeloid differentiation factor 88 (MyD88), a critical component of the MD2/TLR4 signaling pathway, failed to produce significant neuroinflammation and showed improved cognitive ability following surgery [7]. Studies have also shown that MD2 silencing is beneficial for the alleviation of several peripheral inflammation-related diseases, including sepsis, cardiovascular disease and fatty liver disease [8–11]. However, little is known about the role of MD2 in the etiology of PND. Whether MD2 contributes to the pathogenesis of PND and the underlying mechanism have yet to be revealed.

It has been reported that an imbalance in neurotransmitters is a powerful inducer of cognitive impairments [12]. GABA_A_ receptors, which are composed of α1–6, β1–3, γ1–3, ρ1–3, δ, ε, θ, or π subunits, are the major inhibitory neurotransmitter receptors in the brain. It is known that receptors composed of different subunit combinations are localized to different regions and exhibit distinct characteristics [13]. The α1-containing GABA_A_ receptors serve as target for sedative hypnotics [14]. Agonists selective to α2- and/or α3-containing GABA_A_ receptors have been shown to provide an anxiolytic effect [15]. It is reported that α5-containing GABA_A_ (α5GABA_A_) receptors (α5GABA_A_Rs) are mainly located (up to 25%) in the hippocampus of the brain, an area strongly associated with learning and memory in function [15, 16]. α5GABA_A_Rs have been demonstrated to be extrasynaptic [17, 18]. Sustained increase in α5GABA_A_ receptor function impairs memory after anesthesia [19]. α5GABA_A_Rs and their accompanying tonic inhibitory currents have been reported to play an important role in inflammation-induced memory deficits [20]. More importantly, blockade of α5GABA_A_Rs has a neuroprotective effect against anesthesia and surgery-induced cognitive deficits in aged mice [21]. Therefore, the present study aims to investigate the role of MD2 in anesthesia and surgery-induced cognitive deficits and to determine whether α5GABA_A_Rs regulates the function of MD2.

## Materials and methods

### Experimental design

To induce PND, 18-month-old male mice were subjected to laparotomy under 1.4% isoflurane anesthesia, and the Barnes maze was used to assess spatial reference learning and memory. To determine whether MD2 contributes to cognitive decline, we first measured the expression of MD2 in the hippocampus of mice after anesthesia and surgery and then evaluated whether downregulation of MD2 expression by AAV-shMD2 or the MD2-degrading peptide Tat-CIRP-CMA can improve the cognitive ability of surgical mice. To clarify whether MD2 affects the function of α5GABA_A_Rs, we investigated the effects of Tat-CIRP-CMA on the expression of membrane α5GABA_A_Rs in cultured primary neurons and then verified this *in vivo*. In addition, electrophysiology was employed to assess the effect of Tat-CIRP-CMA on tonic currents in CA1 pyramidal neurons in the hippocampus. To further elucidate whether α5GABA_A_Rs regulate the function of MD2, we determined whether inhibiting α5GABA_A_Rs with L-655,708 improves the cognitive ability of surgical mice and whether activating α5GABA_A_Rs with L-838,417 abolishes the protective effect of Tat-CIRP-CMA against anesthesia and surgery-induced cognitive decline.

### Animals

To avoid the variability thought to be caused by the estrous cycle, only male mice were used in this study [22]. Male C57BL/6J mice aged 18 months old were housed in a controlled environment (23 ± 1 °C, 50% humidity, and 12-h light–dark cycle from 7 a.m. to 7 p.m.) with access to food and water ad libitum. All protocols were approved by the Ethics Committee for Animal Experimentation of the Fourth Military Medical University and in accordance with the National Institutes of Health Guide for the Care and Use of Laboratory Animals.

### Laparotomy

Experimental laparotomy was performed as previously described with minor modifications [23]. Briefly, mice were anesthetized with 2.0% isoflurane (Baxter Healthcare, Puerto Rico, USA) for 2 min, and anesthesia was maintained with 1.4% isoflurane. Mice were placed on a heating pad to maintain body temperature between 36.5–37.0 °C. After the hair was shaved and the surgical field was disinfected, an approximately 1-cm median incision was made 0.5–1 cm below the xiphoid, and approximately 5 cm of the small intestine was gently pulled out and then exposed to sterile gauze presoaked with normal saline. After being rubbed for 10 min, the intestine was returned to the abdomen. The muscle and skin were closed layer-by-layer with 5–0 absorbable sutures (Polysorb, COVIDIEN, USA). Finally, 0.1 ml 0.2% lidocaine was injected subcutaneously for postoperative analgesia. The mice were allowed to recover in an incubator at 35 °C for 30 min before being returned to their home cages.

### Behavioral test

The Barnes maze was carried out as described in our previous work [23]. Briefly, the animals were randomly grouped. After 4 consecutive days of training phase (3 trials per mouse on day 5 and 4 trials per mouse on days 6–8 after surgery), the escape compartment was removed, and mice were allowed to move freely for 2 min on day 9 after surgery. All sessions were videotaped and analyzed blindly. The time required to enter the escape compartment and the percentage of time spent in the target quadrant in a 2-min period were calculated to assess the spatial reference learning and memory ability of the mice. All data were analyzed with Anymaze (Stoelting, San Diego, USA).

### Western blot analysis and immunoprecipitation

Hippocampal tissues were homogenized in RIPA lysis buffer comprising 1 mM phosphatase inhibitor, 1 mM protease inhibitor (Roche Applied Science, Basel, Switzerland), and 1 mM PMSF (Beyotime Biotechnology, Shanghai, China) with an ultrasonic processor. The supernatant was collected, loading buffer was added, and the mixture was boiled for 10 min. A plasma membrane protein isolation and cell fraction kit (Invent Biotechnology Inc., MA, USA) was used to extract the membrane proteins. The proteins were separated by SDS-PAGE (Cowin Biosciences, Beijing, China) and then transferred to a methanol-soaked polyvinylidene fluoride membrane (GE healthcare, Boston, USA). The membrane was blocked with 5% milk (BD, New Jersey, USA) for 1 h at room temperature and then incubated with primary antibodies overnight at 4 °C followed by HRP-conjugated secondary antibodies for 1 h at room temperature. The following primary antibodies were used in this study: anti-MD2 (Genetex, GTX85517 1:1000, rabbit), anti-α5GABA_A_R (Abcam, ab242001 1:1000, mouse), anti-tubulin (Abcam, ab7291 1:5000, mouse), anti-β-Actin (Genetex, GTX109639 1:1000, rabbit). The results were analyzed with Image Lab software (Bio-Rad, Hercules, CA, USA).

Hippocampus was processed for immunoprecipitation with anti-MD2 (Genetex, rabbit). Then Western blot detection using anti-α5GABA_A_R (Abcam, mouse) or anti-MD2 was conducted. Of note, we used a light chain-specific IgG antibody to avoid the possible effect of IgG heavy chain on the detection of α5GABA_A_Rs.

### Immunofluorescence

Mice were anesthetized and transcardially perfused with 0.9% saline followed by 4% paraformaldehyde in PBS. The brains were harvested, postfixed in 4% paraformaldehyde overnight at 4 °C, and dehydrated in 30% sucrose in PBS for cryoprotection before being sliced at a thickness of 15 μm on a freezing microtome (Leica, Germany) and mounted onto microscope slides. The sections were incubated with 0.3% Triton X-100 in PBS for 10 min, blocked with 10% donkey serum for 30 min, and then washed with PBS. For double immunofluorescence staining, the sections were incubated with a mixture of anti-MD2 (Abcam, ab24182, 1:100, rabbit) and anti-NeuN (Millipore, MAB377, 1:200, mouse), anti-Iba1 (Abcam, ab5076, 1:200, goat) or anti-GFAP (Abcam, ab4674, 1:200, chicken) antibodies overnight at 4 °C. After three rinses, the sections were incubated with a mixture of Alexa 594-conjugated donkey–anti-rabbit IgG (Invitrogen, 1:400) and Alexa 488-conjugated donkey–anti-mouse IgG (Invitrogen, 1:400), Alexa 488-conjugated donkey–anti-goat IgG (Invitrogen, 1:400), or Alexa 488-conjugated donkey–anti-chicken IgG (Invitrogen, 1:400) antibodies for 2 h at room temperature. The slides were washed and cover-slipped with mounting media containing DAPI (Abcam, ab104139) for observation under a confocal laser scanning microscope (FV10, Olympus, Japan).

### Stereotaxic injection

Mice were anesthetized with isoflurane and secured on a stereotaxic apparatus. After a hole was drilled in the skull, 400 nl of 1 × 10^12^ vg/ml AAV-shMD2 or AAV-shCon (GeneChem, Shanghai, China) was bilaterally injected into the dorsal hippocampus (AP: − 1.50 mm; ML: ± 1.70 mm; DV: − 1.75 mm) at a speed of 50 nl/min. The target sequence was as follows: cgAGGGAATACTATTTCCTAA. The syringe was left for an additional 10 min in place. After injection, the incision was carefully sutured, and the mice were allowed to recover on a heating pad before being returned to their home cages. Laparotomy was performed 3 weeks later. For the lipopolysaccharide (LPS) treatment, LPS (Sigma-Aldrich, L4391) was dissolved in artificial cerebrospinal fluid (ACSF) at the final concentration of 1 mg/ml, and then 2 μl LPS solution was injected into lateral cerebral ventricle (AP: − 0.50 mm; ML: + 1.00 mm; DV: − 1.50 mm) [5].

### Pharmacological treatment

Because CIRP binds to MD2 with high affinity, a peptide Tat-CIRP-CMA (TCM, YGRKKRRQRRR-GRGFSRGGGDRGYGG-KFERQKILDQRFFE, GL Biochem (Shanghai) Ltd, China) that contains Tat transmembrane functional domain (YGRKKRRQRRR), 106-125 domain of CIRP (GRGFSRGGGDRGYGG) and chaperone-mediated autophagy targeting motif (KFERQKILDQRFFE) was synthesized and expected to bypass the blood–brain barrier (BBB) and plasma membrane following peripheral delivery, bind to the endogenous MD2 through peptide–protein interaction, and then direct MD2 degradation through the lysosomal proteolytic machinery [24–26]. The peptide was dissolved in saline and injected via the tail vein at a dose of 20 mg/kg at 24-h intervals for 3 consecutive days starting immediately after the operation. For in vitro experiments, 5 μM Tat-CIRP-CMA was added to the cultured cells for 6 h. L-655,708 or L838,417 (Sigma-Aldrich, Darmstadt, Germany) was dissolved in saline and intraperitoneally administered at a dose of 0.5 mg/kg at 24-h intervals for 3 consecutive days starting immediately after the operation.

### Cultured primary neurons

Pregnant mice were anesthetized and sacrificed on day 14 of pregnancy, and the brain tissue block containing cortices and hippocampus was quickly dissected from the embryos. After trypsinization, a single-cell suspension was obtained and resuspended in neurobasal medium (Gibco, 21103049) comprising 2% B27 supplement (Gibco, 17504-044), 1% penicillin/streptomycin (HyClone, SV30010), and 1% L-glutamate (Gibco, 35050-038). The cells were cultured in six-well plates precoated with poly-D-lysine (50 μg/ml) at 37 °C and 5% CO_2_. The media was changed every 3 days. The neurons were cultured for 14 days before vehicle or Tat-CIRP-CMA treatment.

### Imaging of TCM in the brain *in vivo*

Cyanine 7 monosuccinimidyl ester (Cy7NHS, Xi’an Ruixi Biological Technology Co., Ltd ) (1.5 mg) and Tat-CIRP-CMA (20 mg) were dissolved in 3 ml saline. The mixture solution was stirred on ice for 4 h in the dark, then dialyzed by ddH_2_O to remove excess Cy7NHS. Finally, powder of Cy7 labelled Tat-CIRP-CMA (Cy7-TCM) was obtained by lyophilization. The powder of Cy7NHS was prepared by the same method used as a control. The Cy7-TCM or Cy7NHS was dissolved in saline and injected into 18-month-old mice by tail vein, respectively. The distribution of Cy7-TCM or Cy7NHS in the brain was captured by the IVIS Lumina II *in vivo* optical imaging (Caliper, USA). The mean fluorescence intensity was semi-quantitatively analyzed by a living image system (Caliper, USA) [27]. Subsequently, mice were sacrificed for the observation of the distribution of Cy7-TCM or Cy7NHS in the hippocampus by a confocal laser scanning microscope (FV10, Olympus, Japan).

### Whole-cell electrophysiology

For whole-cell recordings, eighteen-month-old mice brains were removed after anesthetized using isoflurane and immediately submerged in cutting solution comprising (in mM) 225 Sucrose, 2.5 KCl, 1.25 NaH_2_PO_4_, 26 NaHCO_3_, 11 D-glucose, 5 L-Ascorbic acid, 3 Sodium pyruvate, 7 MgSO_4_, and 0.5 CaCl_2_ (pH = 7.4, 290–300 mOsm/kg). Coronal hippocampal sections (300 μm) were cut using a vibratome (Leica VT1200S), kept submerged on ice under oxygenated conditions and then incubated for at least 30 min at 28–30 °C in artificial cerebrospinal fluid (ACSF) comprising (in mM) 122 NaCl, 26 NaHCO_3_, 2.5 KCl, 1.25 NaH_2_PO_4_, 11 D-glucose, 2 MgSO_4_, and 2 CaCl_2_, saturated with 95.0% O_2_/5.0% CO_2_. Then, slices were transferred to a recording chamber and visualized using infrared-differential interference contrast microscopy. Whole-cell recordings were obtained from CA1 pyramidal neurons in the hippocampus using micropipettes prepared from borosilicate glass capillaries using a horizontal puller (P-97, Sutter Instruments) with resistances between 2 and 5 M. The patch electrode solution was composed of (in mM) 140 CsCl, 10 HEPES, 0.2 EGTA, 2 MgCl2, 4 Mg_2_ATP, 0.3 Na_2_GTP, 10 Na_2_-phosphocreatine and 2 QX-314 (pH = 7.3, 290–300 mOsm). Cells were recorded at a holding potential of − 60 mV, and saline + GABA (5 μM) or TCM (5 μM) + GABA (5 μM) was added to ACSF to measure the tonic currents, the GABA_A_ receptor competitive antagonist bicuculline (10 μM) was applied.

### Statistical analysis

All data were analyzed using GraphPad Prism 8.0 Software (GraphPad Prism Co., San Diego, CA, USA). Student’s *t* test (two groups), one-way ANOVA followed by post hoc Dunnett’s or Tukey’s test (multiple groups), and repeat measure two-way ANOVA followed by post hoc Bonferroni’s test (multiple groups at different time point) were used. All data are presented as the mean ± SD. The significance level was set at *P* < 0.05.

## Results

### Anesthesia and surgery induce significant cognitive impairment in aged mice

**Fig. 1:**
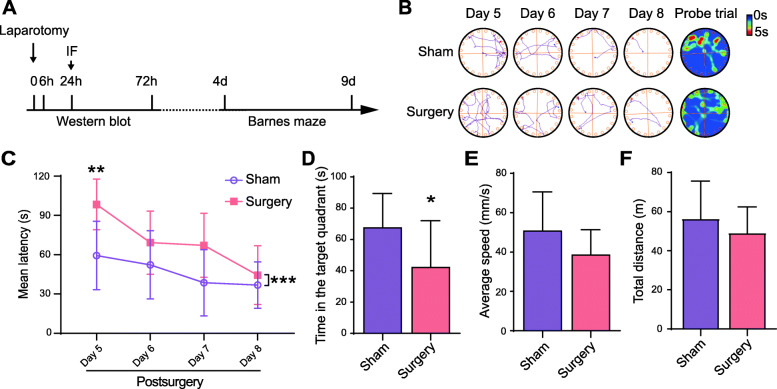
Anesthesia and surgery induce significant cognitive impairment in aged mice. **A** Schematic timeline of western blot analysis and the Barnes maze test. **B** Representative tracks and heat maps of animals in the Barnes maze. **C** Mean latency in the training phase (*F*_(1,18)_ = 18.73, *p =* 0.0004. *post hoc*: day 5 *p* = 0.0059, day 6 *p* = 0.5989, day 7 *p* = 0.0773, day 8 *p* = 0.9999). **D** Time spent in the target quadrant in probe phase (*p* = 0.0394). **E** Average speed in the probe trial (*p* = 0.1087). **F** Total distance in the probe trial (*p* = 0.3378). *n* = 10 each. The data are presented as the mean ± SD, * *p* < 0.05; ** *p* < 0.01; *** *p* < 0.001; compared with the Sham group

To induce PND, aged mice were subjected to laparotomy under 1.4% isoflurane anesthesia, and the Barnes maze was performed 4 days after the operation (Fig. 1A). In the training phase, aged mice subjected to anesthesia and surgery spent more time in searching for the escape compartment than non-surgical controls, indicating a significant anesthesia and surgery-induced decline in spatial reference learning ability (Fig. 1B, C). In the probe phase, the mice spent less time in the target zone after anesthesia and surgery (Fig. 1B, D), indicating a significant impairment of spatial reference memory ability. In addition, no significant difference in the average speed or the total distance was observed between the surgical and nonsurgical groups (Fig. 1E, F).

### Hippocampal MD2 expression is increased in aged mice after anesthesia and surgery

**Fig. 2:**
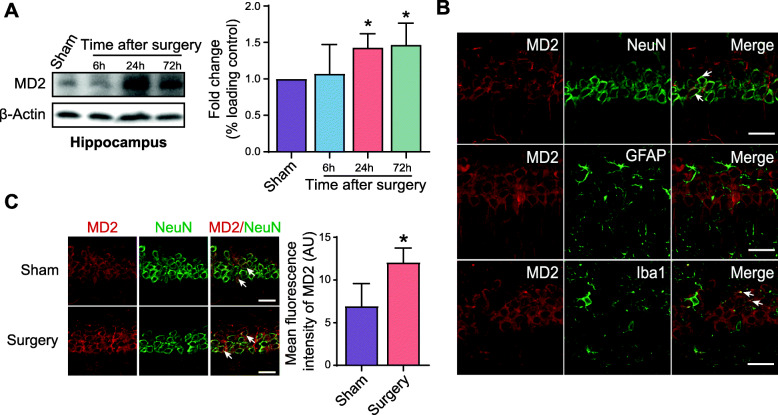
Hippocampal MD2 expression is increased after anesthesia and surgery in aged mice. **A** Expression of hippocampal MD2 at the indicated timepoint after the operation (*F*_(3,20)_ = 4.986, *p* = 0.0096. *p* = 0.0282, Sham vs. 24 h; *p* = 0.0159, Sham vs. 72 h; *n* = 6 each). β-Actin was used as a loading control. **B** Representative immunofluorescence images of MD2 in neurons, astroglia, and microglia in the hippocampus. **C** Representative immunofluorescence images and quantification of neuronal MD2 in the Sham group and Surgery group in the hippocampus (*p* = 0.0470, Sham vs. Surgery; *n* = 3 each). Arrows in the figures indicate the co-localization of two fluorescent substances. The data are presented as the mean ± SD; * *p* < 0.05, compared with the Sham group. Scale bar = 25 μm

We next measured the expression of hippocampal MD2 in mice after anesthesia and surgery. As shown in Fig. 2A, the expression of hippocampal MD2 was stable 6 h after anesthesia and surgery but was obviously increased 1 day after anesthesia and surgery and remained at high levels for a period of 3 days. Notably, in the hippocampus of aged mice, MD2 was prominently expressed in neurons, while a small amount of MD2 expression was also detected in microglia (Fig. 2B). Moreover, the expression of neuronal MD2 increased after anesthesia and surgery (Figs. 1A and 2C).

### Knockdown of MD2 in the hippocampus prevents anesthesia and surgery-induced cognitive decline in aged mice

**Fig. 3:**
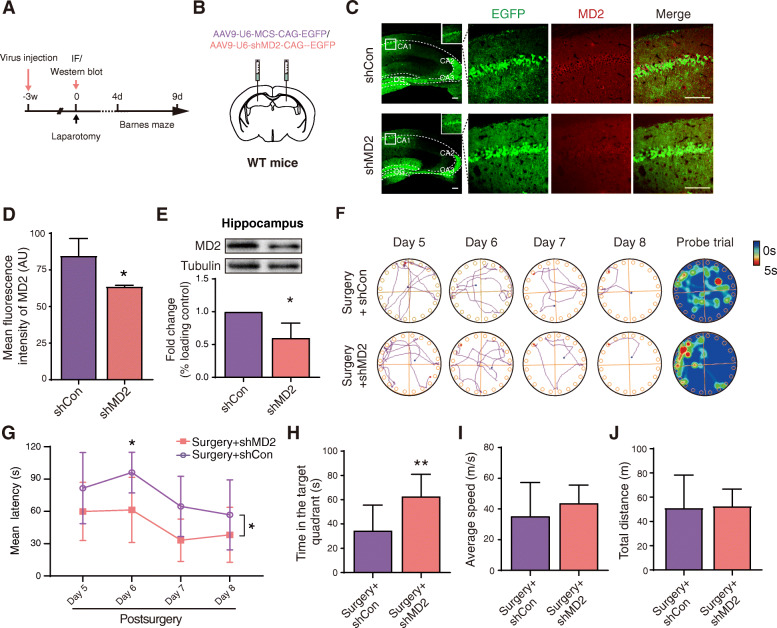
MD2 knockdown by AAV-shMD2 ameliorates anesthesia and surgery-induced cognitive decline. **A** Schematic timeline of virus injection and behavioral test. **B** Schematic diagram of bilateral virus injections. **C** The expression of MD2 in the hippocampus determined by immunofluorescence assay 3 weeks after the virus injection. **D** Statistical analysis of mean immunofluorescence intensity (*p* = 0.0117; *n* = 4 each). Scale bar = 100 μm. **E** Hippocampal MD2 expression determined by western blot analysis (*p* = 0.037; *n* = 3 each). Tubulin was used as a loading control. **F** Representative tracks and heat maps of the mice in the Barnes maze. **G** Mean latency in the training phase (*F*_(1,15)_ = 8.034, *p* = 0.012. *post hoc*: day 5 *p* = 0.430, day 6 *p* = 0.048, day 7 *p* = 0.083, day 8 *p* = 0.661). **H** Time spent in the target zone in the probe phase (*p* = 0.0093). **I** Average speed in the probe phase (*p* = 0.3296). **J** Total distance in the probe phase (*p* = 0.8839). The data are presented as the mean ± SD; * *p* < 0.05, compared with the shCon group or Surgery + shCon group, ** *p* < 0.01, compared with the Surgery + shCon group, *n* = 8 Surgery + shCon, *n* = 9 Surgery + shMD2

To examine whether MD2 contributes to the cognitive decline induced by anesthesia and surgery, we assessed the protective effect of MD2 expression downregulation induced by bilateral injection of AAV-shMD2 into the hippocampus 3 weeks prior to anesthesia and surgery (Fig. 3A, B). In order to verify the effectiveness of AAV-shMD2 in downregulating the expression of MD2, we first observed the expression of EGFP or shMD2-EGFP in the hippocampus 3 weeks following microinjection of the AAV9 and then investigated the efficiency of MD2 knockdown by immunofluorescence assay and western blot assay, respectively. The results showed that either EGFP or shMD2-EGFP was seen in the hippocampus. Meanwhile, the expression of MD2 was downregulated in the hippocampus of mice with AAV-shMD2 (Fig. 3C–E). As predicted, MD2 knockdown mice spent less time in searching for the escape compartment than the control mice in the training phase (Fig. 3F, G), demonstrating that MD2 knockdown improved the spatial reference learning ability of mice subjected to anesthesia and surgery. In the probe phase, MD2 knockdown mice spent more time in the target zone (Fig. 3F, H), indicating that MD2 knockdown improved the spatial reference memory ability of mice subjected to anesthesia and surgery. There was no significant difference in the average speed or the total distance (Fig. 3I, J).

### Tat-CIRP-CMA alleviates cognitive deficits induced by anesthesia and surgery in aged mice

**Fig. 4:**
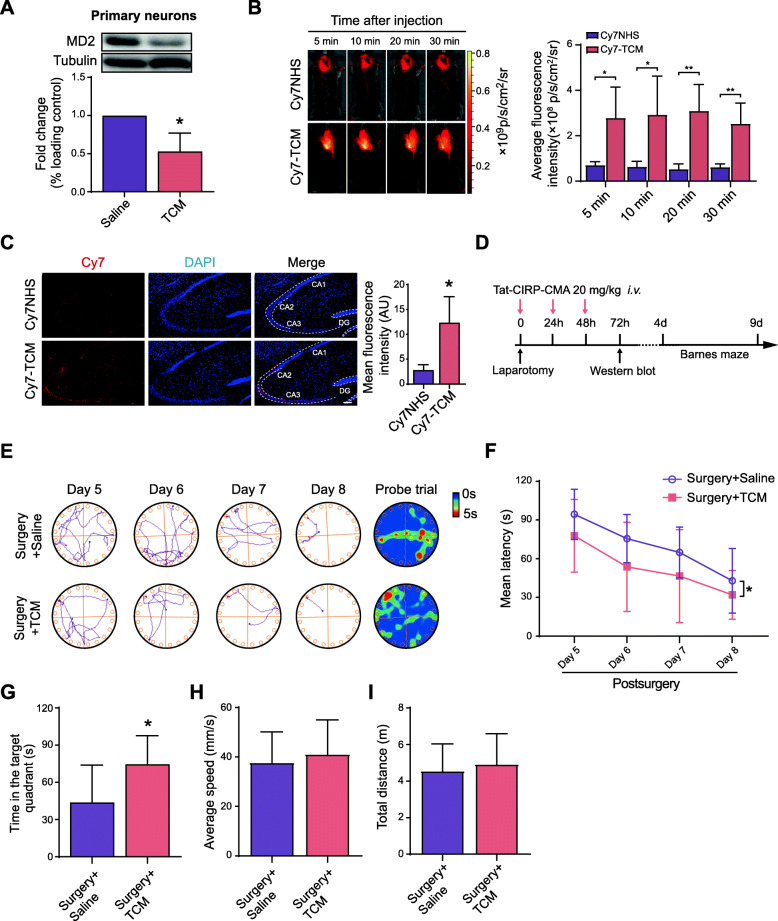
Tat-CIRP-CMA alleviates cognitive decline caused by anesthesia and surgery. **A** Expression of MD2 after TCM treatment in the cultured primary neurons (*p* = 0.0272; *n* = 3 each). Tubulin was used as a loading control. **B** Distribution of Cy7NHS or Cy7-TCM in the brain at 5 min, 10 min, 20 min, and 30 min after peripheral administration (5 min *p* = 0.0231, 10 min *p* = 0.0375, 20 min *p* = 0.0051, 30 min *p* = 0.0059, Cy7NHS vs. Cy7-TCM; *n* = 4 each). **C** Distribution of Cy7NHS or Cy7-TCM in the hippocampus (*p* = 0.0366; *n* = 3 each). Scale bar = 100 μm. **D** Timeline of the TCM treatment. **E** Representative tracks and heat maps of mice in the Barnes maze. **F** Mean latency in the training phase (*F*_(1,13)_ = 6.442, *p* = 0.0247. *post hoc*: day 5 *p* = 0.8663, day 6 *p* = 0.6776, day 7 *p* = 0.9999, day 8 *p* = 0.9999). **G** Time spent in the target zone in the probe phase (*p* = 0.0476). **H** Average speed in the probe phase (*p* = 0.7557). **I** Total distance in the probe phase (*p* = 0.7820). The data are presented as the mean ± SD; * *p* < 0.05, compared with the Saline group, the Surgery + Saline group or the Cy7NHS group; ** *p* < 0.01, compared with the Cy7NHS group. *n* = 8 Surgery + Saline, *n* = 7 Surgery + TCM

Next, we synthesized Tat-CIRP-CMA, a peptide that targets and degrades MD2. The efficiency of MD2 knockdown in the cultured primary neurons was ~ 50%, as shown in Fig. 4A. To examine whether the peptide could cross the blood–brain barrier, the peptide was labelled with Cy7 and injected into 18-month-old mice via tail vein, then, the distribution of Cy7-TCM was captured by the IVIS Lumina II *in vivo* optical imaging. The results showed that after peripheral administration, a certain amount of Cy7-TCM was observed in the brain, demonstrating that the peptide could cross the blood–brain barrier (Fig. 4B). Subsequently, mice were sacrificed for the observation of the distribution of Cy7-TCM or Cy7NHS in the hippocampus. The results showed that the peptide could reach the hippocampus (Fig. 4C). To determine whether Tat-CIRP-CMA can alleviate cognitive decline following anesthesia and surgery, aged mice were intravenously administered Tat-CIRP-CMA for 3 consecutive days after anesthesia and surgery and then subjected to a behavioral test (Fig. 4D). Consistent with the data obtained for mice treated with the MD2 knockdown viruses, mice administered Tat-CIRP-CMA spent less time in searching for the escape compartment than the control mice in the training phase (Fig. 4E, F), indicating that Tat-CIRP-CMA improved the spatial reference learning ability of mice subjected to anesthesia and surgery. In the probe phase, mice administered Tat-CIRP-CMA spent more time in the target zone (Fig. 4E, G), demonstrating that Tat-CIRP-CMA improved the spatial reference memory ability of mice subjected to anesthesia and surgery. Furthermore, there was no significant difference in the average speed or the total distance (Fig. 4H, I). These results suggested that administration of Tat-CIRP-CMA ameliorates cognitive decline after anesthesia and surgery in aged mice.

### Effect of MD2 on the expressions of total α5GABA_A_Rs and membrane α5GABA_A_Rs and the tonic currents in CA1 pyramidal neurons in the hippocampus

**Fig. 5:**
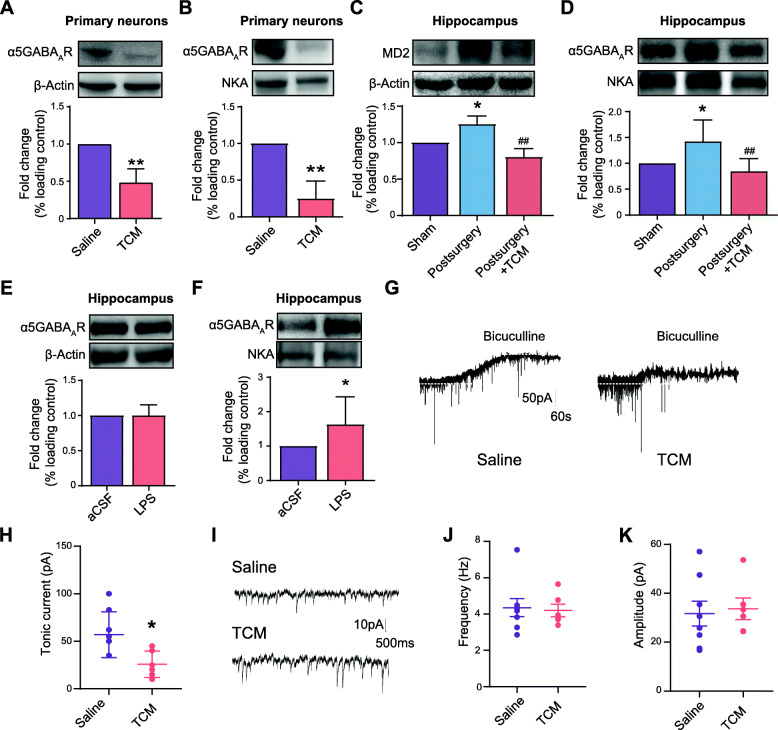
Effect of MD2 on the expressions of total α5GABA_A_Rs and membrane α5GABA_A_Rs and the tonic currents in CA1 pyramidal neurons in the hippocampus. **A** The effect of TCM treatment on the expression of total α5GABA_A_Rs in the cultured primary neurons (*p* = 0.0081; *n* = 3 each). **B** The effect of TCM treatment on the expression of membrane α5GABA_A_Rs in the cultured primary neurons (*p* = 0.0056; *n* = 3 each). **C** The expression of total MD2 in the hippocampus of mice (*F*_(2,6)_ = 17.98, *p* = 0.0029. * *p* = 0.0342, Sham vs. Postsurgery; ^##^ *p* = 0.0024, Postsurgery vs. Postsurgery + TCM; *n* = 3 each). **D** The expression of membrane α5GABA_A_Rs in the hippocampus of mice in the Sham, Postsurgery, and Postsurgery + TCM groups (*F*_(2,15)_ = 6.946, *p* = 0.0073. * *p* = 0.0462, Sham vs. Postsurgery; ^##^ *p* = 0.007, Postsurgery vs. Postsurgery + TCM; *n* = 6 each). Sodium-potassium ATPase (NKA) was used as a loading control. **E** The effect of LPS on the expression of total α5GABA_A_Rs in the hippocampus (*p* = 0.8197; *n* = 4 each). **F** The effect of LPS on membrane α5GABA_A_Rs expression in the hippocampus (*p* = 0.0435; *n* = 8 each). **G**, **H** TCM decreased the tonic currents generated by α5GABA_A_Rs in slices of the hippocampal CA1 subfield (*p* = 0.0158; *n* = 8 Saline, *n* = 6 TCM). **I** TCM had no effect on the sIPSC in slices of the hippocampal CA1 subfield. **J** Frequency of sIPSC (*p* = 0.8165; *n* = 8 Saline, *n* = 6 TCM). **K** Amplitude of sIPSC (*p* = 0.7839; *n* = 8 Saline, *n* = 6 TCM). The data are presented as the mean ± SD; * *p* < 0.05, compared with the Sham group, ACSF group, or Saline group; ** *p* < 0.01, compared with the Saline group; ^##^ *p* < 0.01, compared with the Postsurgery group

The above results showed that MD2 is associated with the occurrence of PND. To illustrate the underlying mechanism, we first investigated the effect of MD2 on the expression of α5GABA_A_Rs *in vitro*. The results showed that after Tat-CIRP-CMA treatment for 6 h, the expression of total α5GABA_A_Rs in cultured primary neurons was significantly decreased (Fig. 5A). Of note, the expression of membrane α5GABA_A_Rs was also remarkably decreased after the TCM treatment (Fig. 5B).

We then performed *in vivo* experiments to verify the effect of MD2 on the expression of α5GABA_A_Rs in the hippocampus. Aged mice were intravenously administered Tat-CIRP-CMA for 3 consecutive days after anesthesia and surgery and then subjected to western blot analysis (Fig. 4D). The results showed that downregulation of MD2 by Tat-CIRP-CMA significantly inhibited the increase in membrane α5GABA_A_Rs expression induced by anesthesia and surgery (Fig. 5C, D). Furthermore, activating MD2 by lipopolysaccharide (LPS) treatment increased the membrane α5GABA_A_Rs, while the total α5GABA_A_Rs were not affected (Fig. 5E, F). The data obtained above indicate a possible role of MD2 in maintaining the content of membrane α5GABA_A_Rs. Being consistent with this, the electrophysiology results showed that Tat-CIRP-CMA inhibited tonic currents (primarily mediated by α5GABA_A_Rs) in CA1 pyramidal neurons in the hippocampus (Fig. 5G, H). Moreover, spontaneous inhibitory synaptic currents (sIPSCs) were recorded and analyzed before bicuculline application to investigate the latent effect of TCM on inhibitory synaptic transmission. The results showed that TCM had no effect on the frequency or the amplitude of sIPSC (Fig. 5I–K), indicating that neither presynaptic GABA release nor postsynaptic function was affected by TCM.

In order to clarify whether MD2 interacted with α5GABA_A_Rs directly in the hippocampus, we performed a Co-IP experiment by using the MD2 antibody; the results showed that there is no interaction between MD2 and α5GABA_A_Rs (Fig. 1).

### Inhibition of α5GABA_A_Rs exerts therapeutic effects against anesthesia and surgery-induced cognitive deficits

**Fig. 6:**
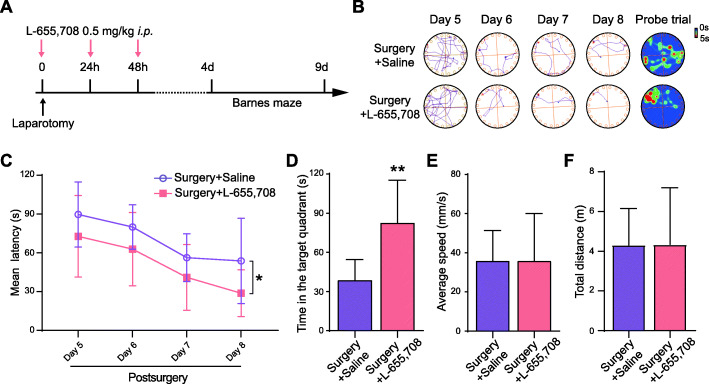
Antagonizing α5GABA_A_Rs reverses anesthesia and surgery-induced cognitive deficits. **A** Timeline of the L-655,708 treatment. **B** Representative tracks and heat maps. **C** Mean latency in the training phase (*F*_(1,13)_ = 6.187, *p* = 0.0272. *post hoc*: day 5 *p* = 0.8236, day 6 *p* = 0.7998, day 7 *p* = 0.9996, day 8 *p* = 0.2488). **D** Time spent in the target zone in the probe day (*p* = 0.0062). **E** Average speed in the probe phase (*p* = 0.9987). **F** Total distance in the probe day (*p* = 0.9843). The data are presented as the mean ± SD; * *p* < 0.05, ** *p* < 0.01, compared with the Surgery + Saline group. *n* = 7 Surgery + Saline, *n* = 8 Surgery + L-655,708

To determine whether inhibition of α5GABA_A_Rs prevents cognitive deficits, L-655,708, an inverse agonist of α5GABA_A_Rs, was intraperitoneally injected into aged mice (Fig. 6A). The results showed that in the training phase, the latency of mice that received L-655,708 injection was obviously shorter than that of mice that received saline (Fig. 6B, C), indicating that inhibition of the α5GABA_A_Rs by L-655,708 improved spatial reference learning ability after anesthesia and surgery. In the probe phase, mice that received L-655,708 injection spent longer time in the target quadrant than mice that received saline (Fig. 6B, D), suggesting that inhibition of the α5GABA_A_Rs by L-655,708 alleviated the spatial reference memory deficits after anesthesia and surgery in aged mice. Furthermore, there was no significant difference in the average speed or the total distance (Fig. 6E, F).

### L-838,417 abrogates the beneficial effects of Tat-CIRP-CMA against anesthesia and surgery-induced cognitive deficits

**Fig. 7:**
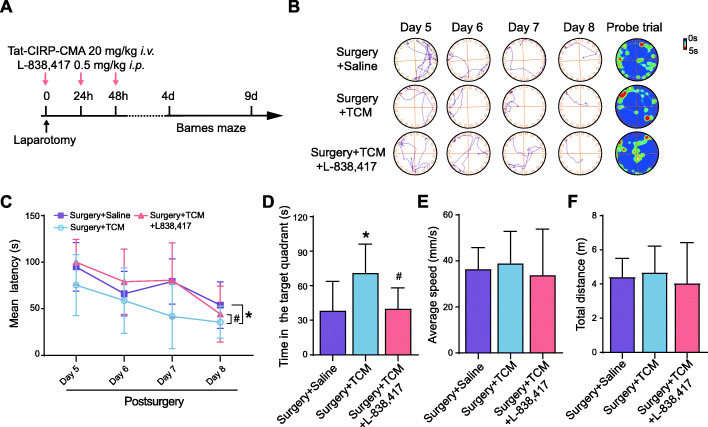
Activating α5GABA_A_Rs abrogates the beneficial effects of Tat-CIRP-CMA against anesthesia and surgery-induced cognitive deficits. **A** Timeline of the L-838,417 treatment. **B** Representative track plots and heat maps. **C** Mean latency in training phase (*F*_(2,20)_ = 4.909, *p* = 0.0184. *post hoc: p =* 0.0286 Surgery + Saline vs. Surgery + TCM, *p =* 0.0443 Surgery + TCM vs. Surgery + L838,417 + TCM). **D** Time spent in the target zone in the probe phase (*F*_(2,20)_ = 5.068, *p =* 0.0166. *post hoc: p =* 0.0254 Surgery + Saline vs. Surgery + TCM, *p =* 0.0422 Surgery + TCM vs. Surgery + L838,417 + TCM). **E** Average speed in the probe phase (*F*_(2,20)_ = 0.2299, *p* = 0.7967. *post hoc*: *p* = 0.9377 Surgery + Saline vs. Surgery + TCM, *p* = 0.7789 Surgery + TCM vs. Surgery + L838,417 + TCM). **F** Total distance in the probe day (*F*_(2,20)_ = 0.2515, *p* = 0.7801. *post hoc*: *p* = 0.9479 Surgery + Saline vs. Surgery + TCM, *p* = 0.7614 Surgery + TCM vs. Surgery + L838,417 + TCM). The data are presented as the mean ± SD; * *p* < 0.05, compared with the Surgery + Saline group; ^#^ *p* < 0.05, compared with the Surgery + TCM group; *n* = 8 Surgery + Saline, *n* = 8 Surgery + TCM, *n* = 7 Surgery + L838,417 + TCM

To assess the causal relationship between MD2 and α5GABA_A_Rs, aged mice were intraperitoneally injected with L-838,417, a partial agonist of α5GABA_A_Rs and intravenously injected with Tat-CIRP-CMA (Fig. 7A) [28, 29]. Behavioral evaluation was performed to investigate whether activation of α5GABA_A_Rs can reverse the therapeutic effects of Tat-CIRP-CMA. The results demonstrated that in the training phase, the latency of mice that received L-838,417 injection and Tat-CIRP-CMA injection was obviously longer than that of mice that received only Tat-CIRP-CMA (Fig. 7B, C), showed that activating α5GABA_A_Rs by L-838,417 abrogated the beneficial effects of Tat-CIRP-CMA against anesthesia and surgery-induced spatial reference learning deficits. In addition, mice that received L-838,417 injection spent less time in the target quadrant than mice that received Tat-CIRP-CMA only (Fig. 7B, D), demonstrating that activation of α5GABA_A_Rs by L-838,417 abrogated the beneficial effects of Tat-CIRP-CMA against anesthesia and surgery-induced spatial reference memory deficits. Furthermore, there was no significant difference in the average speed or the total distance (Fig. 7E, F). These results indicate that α5GABA_A_Rs are involved in MD2-triggered cognitive impairments after anesthesia and surgery.

## Discussion

**Fig. 8:**
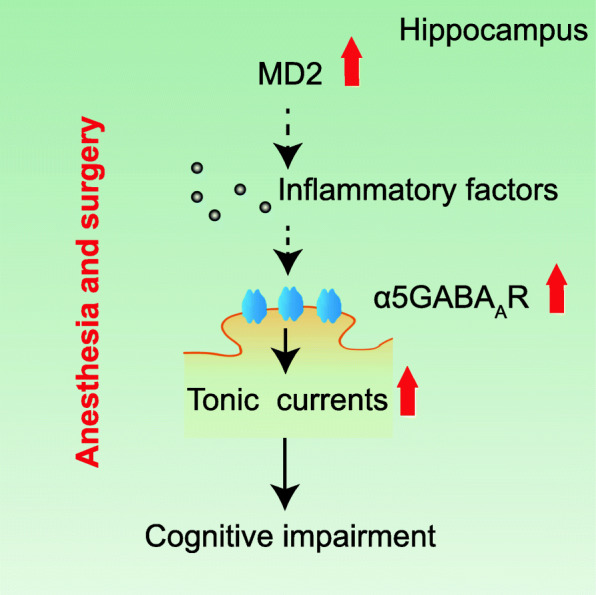
MD2 contributes to the pathogenesis of PND through elevating the expression of α5GABA_A_ receptors and consequent tonic currents in aged mice

In the present study, we found that anesthesia and surgery impaired spatial reference learning and memory ability and elevated MD2 expression in aged mice. MD2 knockdown or administration of the MD2-degrading peptide Tat-CIRP-CMA attenuated cognitive decline. In addition, downregulating MD2 decreased the expression of α5GABA_A_Rs and consequent tonic currents. L-655,708, an inverse agonist of α5GABA_A_Rs, exhibited the protective effects against anesthesia and surgery-induced cognitive impairment, which is consistent with the previous studies showing that L-655,708 mitigates memory deficits after general anesthesia [30, 31]. More importantly, activation of α5GABA_A_Rs abrogated the therapeutic effects of Tat-CIRP-CMA, indicating that α5GABA_A_Rs regulate the function of MD2 in the process of PND. These results demonstrated that MD2 contributed to the pathogenesis of PND through elevating the expression of α5GABA_A_ receptors and consequent tonic currents in aged mice (Fig. 8).

MD2, an essential cofactor of TLR4, has been recognized as an attractive pharmacological target of potent anti-inflammatory agents [32, 33]. For a long time, research on MD2 has been limited to peripheral inflammation-related diseases such as sepsis, diabetes, cardiovascular disease, and fatty liver disease [8, 34–36], and although an increase in the level of TLR4 and downstream inflammatory cascades have been implicated in hippocampus-dependent learning and memory deficits, including PND, the role of MD2 in the pathology of PND has been neglected. For example, TLR4/MyD88 signaling activation by S100A8 promotes neuroinflammation and contributes to tibial fracture surgery-induced cognitive disorders [37]. Inhibition of the hippocampal TLR4 pathway by prophylactic lithium alleviates splenectomy-induced cognitive dysfunction in aged rats [38]. Consistent with previous studies, our results elucidate that activation of MD2 provokes the occurrence and development of PND. Furthermore, our results demonstrate that MD2 degradation by Tat-CIRP-CMA results in the downregulation of α5GABA_A_Rs accompanied by attenuation of tonic currents, suggesting an important role of MD2 in modulating neurotransmitter systems. However, the exact underlying mechanism is still unclear. In fact, we performed Co-IP by using the MD2 antibody to identify the potent interaction between MD2 and α5GABA_A_Rs; unfortunately, the results showed that there is no direct interaction between them (sFig. 1), indicating that the modulation of MD2 on α5GABA_A_Rs is indirect. Further work in aged TLR4^-/-^ mice would clarify whether such regulatory effects depend on TLR4-mediated inflammatory cytokine release. Alternatively, our group’s recent paper published in Science Translational Medicine found that MD2 elicited neuronal apoptosis and necroptosis via the Sam68-related cascade [26]. Whether the Sam68-related cascade is involved in the regulatory effects of MD2 on α5GABA_A_Rs needs further investigation.

MD2 is an initial molecule of inflammatory cascade and typically responds to internal or external stimuli [39]. In this study, we investigated MD2 expression at 6 h, 24 h and 72 h after the operation and then explored whether inhibiting MD2 at an early stage is sufficient to improve the impairments of the whole process of learning and memory in surgical mice by using the Barnes maze. Our results illustrate that the expression of MD2 in the hippocampus is increased after anesthesia and surgery. Although the exact underlying mechanism has not yet been elucidated, we suppose that proinflammatory cytokines released by central immune cells (e.g., glial cells, pericytes, and dendritic cells) and peripheral immune cells (e.g., macrophages, T cells, and B cells) that enter through the disrupted blood–brain barrier (BBB) after laparotomy are likely the main stimuli [40]. In addition, our results showed that hippocampal MD2 was prominently expressed in neurons in both nonsurgical and surgical mice, indicating an important role of neuronal MD2 in PND. However, considering the limitations to antibody specificity, we cannot completely rule out MD2 expression in other cells, such as infiltrating immune cells (macrophages and neutrophils) and glial cells. Further investigation in aged MD2 conditional knockout mice or bone marrow chimeric mice will be useful for demonstrating the importance of MD2 expression in neuronal cells in PND.

In addition, our results showed that MD2 degradation peptide TCM decreased the membrane α5GABA_A_Rs, whereas MD2 activation, induced by LPS stimulation or anesthesia and surgery, elevated the membrane α5GABA_A_Rs (Fig. 5B–E), indicating the possible role of MD2 in maintaining the content of membrane α5GABA_A_Rs. We suppose that once MD2 was degraded after TCM treatment, membrane insertion of α5GABA_A_Rs was inhibited. Consequently, the excess cytoplasmic α5GABA_A_Rs were degraded (Fig. 5A). The underlying mechanism by which MD2 affects the membrane insertion of α5GABA_A_Rs is unclear. Membrane α5GABA_A_Rs expression involves a highly regulated process of synthesis, assembly, endocytosis, and recycling to the membrane or degradation [41]. The role of MD2 in these processes needs further investigation.

Emerging evidences suggest that disturbed function of α5GABA_A_Rs is vital for triggering neurological disorders, and the therapeutic potential of pharmacologic agents targeting α5GABA_A_Rs has been highlighted in recent years [42–44]. Our study showed that α5GABA_A_Rs trigger anesthesia and surgery-induced spatial reference memory impairments, which is consistent with recently published studies showing that α5GABA_A_Rs contribute to anesthesia and surgery-induced conditioned contextual fear memory deficits [21], thus suggesting that α5GABA_A_Rs play an important role in different domains of anesthesia, and surgery-induced hippocampal-dependent cognitive impairments and that α5GABA_A_Rs have great potential as therapeutic targets in PND.

In this study, we showed that L-838,417, a partial agonist for α2-, α3-, and α5-containing GABA_A_ receptors abrogated the therapeutic effects of Tat-CIRP-CMA on perioperative neurocognitive disorder [28, 29]. Considering that α2- and α3-containing GABA_A_ receptors are believed to be involved in the anxiolytic effect, whereas α5 subunits play a role in cognitive performance [15, 16], we deduce that the effect of L-838,417 on PND seems to be mainly due to its activating effect on α5-containing GABA_A_ receptors. However, it needs to be emphasized that we cannot rule out the possible effect of L-838,417 on α2- and α3-containing GABA_A_ receptors in this regard. A systematic study is needed to further understand the role(s) of α2 and/or α3 in the occurrence of PND.

PND has been recognized for more than 100 years, and many studies aiming to elucidate the pathogenesis of PND from different perspectives, including impaired synaptic function, neuroinflammation, oxidative stress in the central nervous system, and the microbiota–gut-brain axis, have been performed [45–48]. Although these studies have helped to exploit pharmacologic agents to treat PND, few pharmacologic agents have been developed. Further progress in clinical translation is needed. MD2 is a protein containing only 160 amino acids and is a promising drug target [49]. In this study, a 40-residue peptide is synthesized to target and degrade MD2. This peptide was shown to be effective against anesthesia and surgery-induced memory impairments, exhibiting great potential for clinical translation. Regrettably, only male mice were used in this study to avoid the possible bias caused by sex difference. A longitudinal cohort analysis including 1033 participants showed that although there were no significant sex differences in postoperative cognitive outcomes, APOE4+ older men may be more vulnerable to PND than APOE4+ older women, indicating the important role of sex and genetic variables on PND [50]. In addition, the estradiol’s effect on neuronal systems and cognitive function has been widely reported [51]. The potential role of TCM in females might be worth being investigated in future.

## Conclusions

In conclusion, our findings suggest that upregulated MD2 induced by anesthesia and surgery contributes to the occurrence of PND by regulating α5GABA_A_ receptors in aged mice, and Tat-CIRP-CMA is a promising neuroprotectant against anesthesia and surgery-induced cognitive deficits.

## Supplementary Information

**Additional file 1: Supplementary Fig. 1.** Co-immunoprecipitation of MD2 and α5GABA_A_Rs.

## Data Availability

The data supporting the findings of this study are presented within the manuscript.

## Abbreviations

PND: Perioperative neurocognitive disorder
MD2: Myeloid differentiation factor 2
TLR4: Toll-like receptor 4
GABA: γ-aminobutyric acid
α5GABA_A_R: α5 subunit-containing GABA_A_ receptor
BBB: Blood–brain barrier
NeuN: Neuron-specific nuclear protein
Iba1: Ionized calcium binding adapter molecule 1
GFAP: Glial fibrillary acidic protein
NKA: Sodium-potassium ATPase
IP-MS: Immunoprecipitation–mass spectrometry
ACSF: Artificial cerebrospinal fluid

## References

[1] Gou RY, Hshieh TT, Marcantonio ER, Cooper Z, Jones RN, Travison TG, Fong TG, Abdeen A, Lange J, Earp B, Schmitt EM, Leslie DL, Inouye SK, SAGES Study Group (2021). One-year Medicare costs associated with delirium in older patients undergoing major elective surgery. JAMA Surg.

[2] Saxena S, Maze M (2018). Impact on the brain of the inflammatory response to surgery. Presse Med.

[3] Yang Y, Hu Y, Zhou Y, Liang T, Tang H, Ju H, et al. Lys694Arg polymorphism leads to blunted responses to LPS by interfering TLR4 with recruitment of MyD88. Innate Immun. 2020;1753425920927479. 10.1177/1753425920927479 PMC8504268 32513051

[4] Eisenstein TK (2019). The role of opioid receptors in immune system function. Front Immunol.

[5] Zhang XY, Cao JB, Zhang LM, Li YF, Mi WD (2015). Deferoxamine attenuates lipopolysaccharide-induced neuroinflammation and memory impairment in mice. J Neuroinflammation.

[6] Park BS, Song DH, Kim HM, Choi BS, Lee H, Lee JO (2009). The structural basis of lipopolysaccharide recognition by the TLR4-MD-2 complex. Nature.

[7] Terrando N, Monaco C, Ma D, Foxwell BM, Feldmann M, Maze M (2010). Tumor necrosis factor-alpha triggers a cytokine cascade yielding postoperative cognitive decline. Proc Natl Acad Sci U S A.

[8] Zhang Y, Wu B, Zhang H, Ge X, Ying S, Hu M, Li W, Huang Y, Wang L, Chen C, Shan X, Liang G (2018). Inhibition of MD2-dependent inflammation attenuates the progression of non-alcoholic fatty liver disease. J Cell Mol Med.

[9] Chen G, Xiao B, Chen L, Bai B, Zhang Y, Xu Z, Fu L, Liu Z, Li X, Zhao Y, Liang G (2017). Discovery of new MD2-targeted anti-inflammatory compounds for the treatment of sepsis and acute lung injury. Eur J Med Chem.

[10] Wang Y, Qian Y, Fang Q, Zhong P, Li W, Wang L, Fu W, Zhang Y, Xu Z, Li X, Liang G (2017). Saturated palmitic acid induces myocardial inflammatory injuries through direct binding to TLR4 accessory protein MD2. Nat Commun.

[11] Sumneang N, Apaijai N, Chattipakorn SC, Chattipakorn N (2020). Myeloid differentiation factor 2 in the heart: bench to bedside evidence for potential clinical benefits?. Pharmacol Res.

[12] Kandimalla R, Reddy PH (2017). Therapeutics of neurotransmitters in Alzheimer's disease. J Alzheimers Dis.

[13] Rudolph U, Knoflach F (2011). Beyond classical benzodiazepines: novel therapeutic potential of GABAA receptor subtypes. Nat Rev Drug Discov.

[14] McKernan RM, Rosahl TW, Reynolds DS, Sur C, Wafford KA, Atack JR, Farrar S, Myers J, Cook G, Ferris P (2000). Sedative but not anxiolytic properties of benzodiazepines are mediated by the GABA(A) receptor alpha1 subtype. Nat Neurosci.

[15] Mohamad FH, Has ATC (2019). The alpha5-containing GABAA receptors-a brief summary. J Mol Neurosci.

[16] Rudolph U, Mohler H (2006). GABA-based therapeutic approaches: GABAA receptor subtype functions. Curr Opin Pharmacol.

[17] Hannan S, Minere M, Harris J, Izquierdo P, Thomas P, Tench B, Smart TG (2020). GABA(A)R isoform and subunit structural motifs determine synaptic and extrasynaptic receptor localisation. Neuropharmacology.

[18] Möhler H (2006). GABA(A) receptor diversity and pharmacology. Cell Tissue Res.

[19] Zurek AA, Yu J, Wang DS, Haffey SC, Bridgwater EM, Penna A, Lecker I, Lei G, Chang T, Salter EW, Orser BA (2014). Sustained increase in alpha5GABAA receptor function impairs memory after anesthesia. J Clin Invest.

[20] Wang DS, Zurek AA, Lecker I, Yu J, Abramian AM, Avramescu S, Davies PA, Moss SJ, Lu WY, Orser BA (2012). Memory deficits induced by inflammation are regulated by alpha5-subunit-containing GABAA receptors. Cell Rep.

[21] Zhang W, Xiong BR, Zhang LQ, Huang X, Zhou WC, Zou Q, Manyande A, Wang J, Tian XB, Tian YK (2020). Disruption of the GABAergic system contributes to the development of perioperative neurocognitive disorders after anesthesia and surgery in aged mice. CNS Neurosci Ther.

[22] Milad MR, Igoe SA, Lebron-Milad K, Novales JE (2009). Estrous cycle phase and gonadal hormones influence conditioned fear extinction. Neuroscience.

[23] Zhao G, Deng J, Shen Y, Zhang P, Dong H, Xie Z, Xiong L (2019). Hyperhomocysteinemia is key for increased susceptibility to PND in aged mice. Ann Clin Transl Neurol.

[24] Qiang X, Yang WL, Wu R, Zhou M, Jacob A, Dong W, Kuncewitch M, Ji Y, Yang H, Wang H, Fujita J, Nicastro J, Coppa GF, Tracey KJ, Wang P (2013). Cold-inducible RNA-binding protein (CIRP) triggers inflammatory responses in hemorrhagic shock and sepsis. Nat Med.

[25] Fan X, Jin WY, Lu J, Wang J, Wang YT (2014). Rapid and reversible knockdown of endogenous proteins by peptide-directed lysosomal degradation. Nat Neurosci.

[26] Fang Z, Wu D, Deng J, Yang Q, Zhang X, Chen J, Wang S, Hu S, Hou W, Ning S, Ding Y, Fan Z, Jiang Z, Kang J, Liu Y, Miao J, Ji X, Dong H, Xiong L (2021). An MD2-perturbing peptide has therapeutic effects in rodent and rhesus monkey models of stroke. Sci Transl Med.

[27] Qiao S, Cheng Y, Liu M, Ji Q, Zhang B, Mei Q, Liu D, Zhou S (2021). Chemoattractants driven and microglia based biomimetic nanoparticle treating TMZ-resistant glioblastoma multiforme. J Control Release.

[28] Bravo-Hernandez M, Corleto JA, Barragan-Iglesias P, Gonzalez-Ramirez R, Pineda-Farias JB, Felix R, Calcutt NA, Delgado-Lezama R, Marsala M, Granados-Soto V (2016). The alpha5 subunit containing GABAA receptors contribute to chronic pain. Pain.

[29] Rowlett JK, Platt DM, Lelas S, Atack JR, Dawson GR (2005). Different GABAA receptor subtypes mediate the anxiolytic, abuse-related, and motor effects of benzodiazepine-like drugs in primates. Proc Natl Acad Sci U S A.

[30] Martin LJ, Oh GH, Orser BA (2009). Etomidate targets alpha5 gamma-aminobutyric acid subtype A receptors to regulate synaptic plasticity and memory blockade. Anesthesiology.

[31] Saab BJ, Maclean AJ, Kanisek M, Zurek AA, Martin LJ, Roder JC, Orser BA (2010). Short-term memory impairment after isoflurane in mice is prevented by the alpha5 gamma-aminobutyric acid type A receptor inverse agonist L-655,708. Anesthesiology.

[32] Qiu Y, Xiao Z, Wang Y, Zhang D, Zhang W, Wang G, Chen W, Liang G, Li X, Zhang Y, Liu Z (2019). Optimization and anti-inflammatory evaluation of methyl gallate derivatives as a myeloid differentiation protein 2 inhibitor. Bioorg Med Chem.

[33] Chen L, Fu W, Zheng L, Wang Y, Liang G (2018). Recent progress in the discovery of myeloid differentiation 2 (MD2) modulators for inflammatory diseases. Drug Discov Today.

[34] Zhang Y, Chen H, Zhang W, Cai Y, Shan P, Wu D, Zhang B, Liu H, Khan ZA, Liang G (1866). Arachidonic acid inhibits inflammatory responses by binding to myeloid differentiation factor-2 (MD2) and preventing MD2/Toll-like receptor 4 signaling activation. Biochim Biophys Acta Mol Basis Dis.

[35] de Oliveira AA, Faustino J, Webb RC, Nunes KP (2020). Blockade of the TLR4-MD2 complex lowers blood pressure and improves vascular function in a murine model of type 1 diabetes. Sci Rep.

[36] Wang Y, Luo W, Han J, Khan ZA, Fang Q, Jin Y, Chen X, Zhang Y, Wang M, Qian J, Huang W, Lum H, Wu G, Liang G (2020). MD2 activation by direct AGE interaction drives inflammatory diabetic cardiomyopathy. Nat Commun.

[37] Lu SM, Yu CJ, Liu YH, Dong HQ, Zhang X, Zhang SS, Hu LQ, Zhang F, Qian YN, Gui B (2015). S100A8 contributes to postoperative cognitive dysfunction in mice undergoing tibial fracture surgery by activating the TLR4/MyD88 pathway. Brain Behav Immun.

[38] Lu SM, Gui B, Dong HQ, Zhang X, Zhang SS, Hu LQ, Liu HL, Sun J, Qian YN (2015). Prophylactic lithium alleviates splenectomy-induced cognitive dysfunction possibly by inhibiting hippocampal TLR4 activation in aged rats. Brain Res Bull.

[39] Tandon A, Harioudh MK, Ishrat N, Tripathi AK, Srivastava S, Ghosh JK (2018). An MD2-derived peptide promotes LPS aggregation, facilitates its internalization in THP-1 cells, and inhibits LPS-induced pro-inflammatory responses. Cell Mol Life Sci.

[40] Oo TT, Pratchayasakul W, Chattipakorn N, Chattipakorn SC (2020). Potential roles of myeloid differentiation factor 2 on neuroinflammation and its possible interventions. Mol Neurobiol.

[41] Kumar S, Fleming RL, Morrow AL (2004). Ethanol regulation of gamma-aminobutyric acid A receptors: genomic and nongenomic mechanisms. Pharmacol Ther.

[42] Zurek AA, Kemp SW, Aga Z, Walker S, Milenkovic M, Ramsey AJ, Sibille E, Scherer SW, Orser BA (2016). α5GABAA receptor deficiency causes autism-like behaviors. Ann Clin Transl Neurol.

[43] Prevot TD, Sumitomo A, Tomoda T, Knutson DE, Li G, Mondal P, Banasr M, Cook JM, Sibille E (2021). Reversal of age-related neuronal atrophy by α5-GABAA receptor positive allosteric modulation. Cereb Cortex.

[44] Engin E, Sigal M, Benke D, Zeller A, Rudolph U (2020). Bidirectional regulation of distinct memory domains by α5-subunit-containing GABA(A) receptors in CA1 pyramidal neurons. Learn Mem.

[45] Yin L, Gao S, Li C (2020). Exogenous hydrogen sulfide alleviates surgery-induced neuroinflammatory cognitive impairment in adult mice by inhibiting NO signaling. BMC Anesthesiol.

[46] Wang T, Zhu H, Hou Y, Gu W, Wu H, Luan Y, Xiao C, Zhou C (2019). Galantamine reversed early postoperative cognitive deficit via alleviating inflammation and enhancing synaptic transmission in mouse hippocampus. Eur J Pharmacol.

[47] Wei P, Yang F, Zheng Q, Tang W, Li J (2019). The potential role of the NLRP3 inflammasome activation as a link between mitochondria ROS generation and neuroinflammation in postoperative cognitive dysfunction. Front Cell Neurosci.

[48] Jiang XL, Gu XY, Zhou XX, Chen XM, Zhang X, Yang YT, Qin Y, Shen L, Yu WF, Su DS (2019). Intestinal dysbacteriosis mediates the reference memory deficit induced by anaesthesia/surgery in aged mice. Brain Behav Immun.

[49] Visintin A, Iliev DB, Monks BG, Halmen KA, Golenbock DT (2006). MD-2. Immunobiology.

[50] Schenning KJ, Murchison CF, Mattek NC, Kaye JA, Quinn JF (2019). Sex and genetic differences in postoperative cognitive dysfunction: a longitudinal cohort analysis. Biol Sex Differ.

[51] Luine VN (2014). Estradiol and cognitive function: past, present and future. Horm Behav.

