# Supplementary figures and images for: MD2 contributes to the pathogenesis of perioperative neurocognitive disorder via the regulation of α5GABAA receptors in aged mice
Source: J Neuroinflammation. 2021 Sep 16;18:204. doi: 10.1186/s12974-021-02246-4 (PMC8444589; doi:10.1186/s12974-021-02246-4)

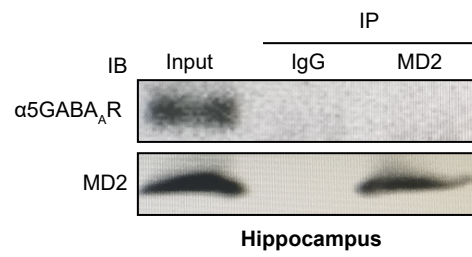

Supplement: Supplementary file 1 — Additional file 1: Supplementary Fig. 1. Co-immunoprecipitation of MD2 and α5GABAARs. [file 12974_2021_2246_MOESM1_ESM.pdf]
